# Genes, cells, and biobanks: Yes, there’s still a consent problem

**DOI:** 10.1371/journal.pbio.2002654

**Published:** 2017-07-25

**Authors:** Timothy Caulfield, Blake Murdoch

**Affiliations:** Health Law Institute, Faculty of Law, University of Alberta, Edmonton, Alberta, Canada

## Abstract

From a research perspective, the interest in biobanking continues to intensify. Governments and industry have invested heavily in biobanks, as exemplified by initiatives like the United Kingdom Biobank and United States' Precision Medicine Initiative. But despite this enthusiasm, many profound legal and ethical challenges remain unresolved. Indeed, there continues to be disagreements about how best to obtain consent and the degree and nature of control that research participants retain over donated samples and health information. Emerging social trends—including concerns about commercialization and perceived rights of continuing control (“biorights”)—seem likely to intensify these issues.

## Introduction

Driven by advances in genetics, information technology, and cell-line research, interest in the collection and analysis of human biological material continues to intensify. Over the past few decades, there has been a proliferation of biobanks—both large and small [1]—that link tissue and genetic information to a host of other forms of health and personal data. Indeed, biobanking and related research methods have been characterized as an essential and potentially “revolutionizing” approach to biomedical research [2]. In 2009, for example, a *Time Magazine* cover story framed biobanking as a “world changing” idea [3]. Since then, the enthusiasm has not diminished. Governments and industries throughout the world have invested heavily in biobanking [4]. This is perhaps best exemplified by former President Barack Obama’s championing of the Precision Medicine Initiative [5], which, among other things, includes the creation of a large national biobank [6].

In addition to the excitement flowing from the scientific and medical potential of biobanking, there has been a great deal of controversy. Much of the conflict has centred on issues of consent and the control of tissue samples. Because of the large number of participants (UK Biobank, for example, recruited 500,000 individuals between 2006−2010 [7]), the involvement of multiple researchers, and the long-term nature of the initiatives, traditional models of consent are considered impractical, if not impossible. As such, biobanks have adopted consent strategies that deviate from traditional legal norms, most often involving the use of some form of broad or open consent [8]. But despite the mass utilization of these approaches, there remains no consensus as to their legal and ethical appropriateness. A 2012 analysis of relevant literature, research ethics policies, and public perception data found that, aside from within the biobanking research community, there is no consensus on the consent issue [9]. And there is little reason to think that a consensus will coalesce in the future, despite attempts by some jurisdictions, including the US and the European Union, to craft relevant policy [10,11].

We live in a fascinating and potentially precarious time. On the one hand, there continues to be escalating growth, investment and excitement surrounding biobanking. Across the globe, millions of individuals have been recruited to participate in these complex, expensive research platforms. And the potential scientific and health benefits are undoubtedly real. On the other hand, there remains a deep lack of clarity around basic legal and ethical principles. The public is supportive, but that support appears tentative and conditional on the maintenance of trust. The bottom line: the international research community has built a massive and diverse research infrastructure on a foundation that has the potential, however slight, to collapse, in bits or altogether. Those most involved in the research—that is, those involved with the collection of samples and the establishment and administration of biobanks—appear to be operating under the belief that the issues associated with the law and public opinion are either settled or manageable within existing frameworks. Here, we seek to highlight how wrong such assumptions are. What is needed is real policy reform. We believe this would benefit from more explicit recognition of the vast disconnect between the current practices and the realities of the law, research ethics, and public perceptions.

Of course, concerns about consent and ownership are hardly new. On the contrary, biobanking has gained support and flourished despite the ongoing and frequently articulated apprehension surrounding issues like consent and ownership. However, there are emerging social trends and technological developments—several of which we review below—that have heightened the need for increased clarity in the context of consent policy.

### Unsettled law

One would expect that with so much activity and such broad-based investment, the law relevant to biobanking would be relatively settled, especially for something as fundamental to the research ethics process. In fact, there remains a great deal of uncertainty regarding, inter alia, the ownership of samples [12–14] and what type of consent is legally appropriate. A recent review of Canadian law by a team at McGill University, for example, concluded that while broad consent is ubiquitous, it “does not seem to fulfill legal and ethical informational requirements” [15]. It has been suggested that emerging regulations in Europe [16] dictate that “consent must be ‘specific and informed’” [11]. A 2017 revision of the US Common Rule, the country’s national research ethics guideline, explicitly endorses the use of broad consent in specific situations [17,18,19], but this policy change does not resolve debate about whether such an approach is appropriate; as noted by a commentator, “potential subjects cannot be informed of the specific risks and benefits of research because the biobanks do not know what those risks or benefits may be” [20]. It has been suggested, for example, that the new regulations—which disappointed patient advocacy groups [21]—reflect more the power of the research institutions’ lobby than a conceptually and legally coherent policy change [22].

Here, we offer no speculation about what the law ought to be (a topic one of us has covered elsewhere [23]). Our key point is straightforward: despite decades of debate and a huge amount of public and private investment in biobanking, there is still a great deal of ambiguity and uncertainty regarding the issues associated with participant control of specimens and health information [24]. Yet despite this reality, biobanks throughout the world have forged ahead using various alternatives to traditional models of consent and governance structures [9,20,25]. Given that this research method seems unlikely to go away anytime soon, it is worthwhile to reflect on the building tension between the enthusiasm for biobanks and the need for a robust and conceptually consistent framework for consent.

### Public perception

A great deal of public perception research has been done on the issues associated with biobanking. While there are few uniform messages that flow from this body of data, one thing can be said with certainty: there is no consensus on how to handle consent. For example, a 2016 study in the US found that “nearly 44% of our nationally representative sample found blanket consent unacceptable and 38% felt it was, in fact, the worst in a range of consent policy options” [26]. Other research has also found that a significant portion of the public is not keen on the use of broad, open, or blanket consent if other options are available [27,28]. Indeed, a 2015 systematic review of the available data concluded that “the most notable finding is that many people do not favor broad consent for either research itself or for research and subsequent wide data sharing” [29]. Yes, research has found that many in the public find broad consent acceptable [30], but this does not mean that it is the preference or that the approach is without controversy. Naturally, a broad or general consent approach is clearly preferred by biobank researchers [31]. But, as reported in a 2015 survey, the scientific community also “does not believe there to be a consensus on consent type” [32].

When it comes to the public’s perception of consent in the context of biobanks, the most that can be said is that it remains unsettled [27,28,29,32] and there seems little reason to think a unified position will emerge. In fact, as we will see, there are several social trends that suggest the environment may become even more confused.

### Perceived rights of control

The growing interest in the concept of biorights could be particularly disruptive. This refers to the idea that research participants have an ongoing right to control their research samples, to benefit directly from the research, and/or to be financially compensated for their contribution [33,34]. While the existing public perception research suggests that this view is likely held by only a minority—most in the public remain willing to contribute to biobanks and similar initiatives [35,30]—it would not take many individuals supportive of the idea of biorights to complicate the consent process and the concomitant public discourse, as we have seen with a number of related controversies [36]. For example, the infamous case of Henrietta Lacks, who was the source of the HeLa cell line, continues to stir controversy. Members of the family are now seeking financial compensation for the cells [37], and the story of Henrietta Lacks is being made into a movie starring Oprah Winfrey [38].

Ironically, the growing interest in a perceived right of control may be due, in part, to exaggerated claims of benefit flowing from both the research community and the popular press. A 2014 study found that many of the representations of biobanks in the popular press are “hyped”—that is, they contain exaggeration of both the near future benefits and a minimization of risks and limitations [39]. And, of course, genetic research more broadly has been the subject of a great deal of overpromise [40]. The same can be said for stem cell research [41].

All of this positive coverage—accurate or not—may contribute to the perception that genetic information and human cells are especially sensitive, valuable, and worthy of unique protections and individual control. Headlines like “Could we one day make babies from only skin cells?” [42], “Cell Line Development Market worth $3.96 Billion by 2019” [43] and “Human Biobanking Ownership–Market to Witness a value of $37.1 Billion by 2020” [44] add to the impression that there may be many reasons to retain a strong right of control over donated research samples. A 2016 American news item—which also offered yet another estimation of value, suggesting that biological samples collected by researchers might generate “$23 billion in revenue by 2018”—quoted a research participant who demanded and received payment for her biological materials [33]. The research participant suggested “there has been an over-assumption and a gross expectation of patient altruism” [33]. This kind of reaction fits well with a prediction made by Stanford law professor Hank Greely in 2010: “As more and more people find out what can be done—or is being done—with their health information, their family histories, and their DNA, the pressure for change should grow” [45].

When thinking about the forces that drive social controversy, it does not matter whether these views regarding biorights and the value of genetic information are justified. In fact, there are reasons to dispute the idea that genetic information is uniquely valuable [46] and, as one of us has argued elsewhere, public perception should not necessarily drive policy [47]. Yet, it often does [48]. Public perception can also drive public debate and can serve as a barometer of future social controversy.

### Public trust and commercialization

Surveys have consistently found that the public places a great deal of trust in researchers and research institutions. However, public perception studies have also found that trust can be easily lost. Any involvement with industry, for example, erodes public confidence in the biobanking enterprise [49]. A 2012 survey of 1,201 people in Alberta, Canada found that 45.1% had a “great deal” of trust in university-funded researchers, but only 19.5% felt the same way about university researchers who received funding from industry [50]. The number drops to 6% for biobanking research done by industry [50]. Commercial interests seem to have the same impact on how the public perceives the acceptability of various forms of consent. A 2016 study found that a majority of individuals (68%) were willing to provide a blanket consent, but that drops to 55% if their specimens might be used “to develop patents and earn profits for commercial companies” [51,52].

One of the key goals of biobanks is to produce technologies and drugs that will be used in the healthcare context. Industry will inevitably be involved, particularly as the work gets closer to clinical application. As argued in a 2015 commentary, “ultimately, the success of future biobanks will rely greatly on the success of public-private partnerships” [53]. In addition, high maintenance costs mean that many biobanks must turn to industry for funding support, which some fear could affect the independence and integrity of research, or result in changes in the use of data or samples that are inconsistent with existing consents [24]. The inevitability of increased industry involvement creates the potential to erode public trust and to intensify the challenges associated with consent and sample ownership [24].

And this push to commercialize can also have a direct impact on the consent process, heightening the need for specific consent or, at least, specific disclosures about commercialization and industry involvement. The importance of informing participants about commercialization and industry activities has been recognized as a key part of the consent process and something participants want to know [24,54]. Indeed, studies have found that “information about sponsoring of biobank research by pharmaceutical industry was associated negatively with a preference for broad consent” [55]. It is also worth noting that Article 26 of the European Union’s Directive 98/44/EC on the legal protection of biotechnological inventions states that before issuing a patent, it should be established that free and informed consent has been obtained from individuals who have contributed the relevant biological material [56]. In sum, increasing commercialization pressure seems likely to intensify the already complex consent and control issues associated with biobanks.

### Privacy and discrimination concerns and controversies

Public interest in maintaining control over biobanked information and samples may be heightened by highly publicized instances of the mishandling of private health information. Data breaches involving confidential medical information are increasing in number [57]. For example, in Georgia, the Emory Clinic experienced unauthorized data access in March 2017 that resulted in 79,930 individuals’ personal information being compromised [58]. Similarly, Alberta Health Services recently experienced a privacy breach involving the confidential health information of 12,848 individuals [59]. These stories fuel growing public concern over the protection of personal health information [60,61]. Survey data indicate that members of the public worry about loss of control over their data, unauthorized use of it, and potential associated harms, including the possibility that health information could be wrongly used to inform discriminatory corporate, institutional, or public policies [60,62].

Several issues compound these privacy concerns in the context of biobanking. First, there is survey evidence indicating biobank participants are sometimes not fully aware of the confidentiality risks inherent to their participation [63]. Given research finding that privacy issues are important to biobank participants [64], any mishandling of the disclosure process by biobanks could have a severe adverse impact on public trust. Second, rightly or not, the public believes genetic information is particularly sensitive [65–68]—a perception likely fuelled by overly optimistic media portrayals and cautionary stories about the potential for genetic discrimination [69]. As a result, any privacy issue that engages genetics is likely to heighten the public’s desire to maintain control. Finally, the ability to pull information from cells, tissue, and genetic material has advanced rapidly over the past few decades. Indeed, the sequencing of entire genomes has become increasingly inexpensive and routine. This digitization of tissue, cells, and genetic data means that the line between health information (or health records) and tissue has largely disappeared [70,71].

### Technology and consent opportunities

The core justification for a move away from specific, case-by-case consent is that it is inefficient and expensive. But as information technology moves forward, that justification is weakened [72,73]. New tools are being developed to easily and inexpensively allow donors and participants to remain in constant contact with researchers and biobanks [74]. These tools can be used to allow participants whose health information and biological materials were collected by biobanks before the age of majority to reconsent or withdraw consent as adults, a practice which some suggest is an ethical necessity [75]. Indeed, the use of electronic consent systems is “a feasible and potentially game-changing strategy” for large research studies that depend on patient recruitment [76]. It is possible the public may increasingly view broad consent as inappropriate in light of the ability to use technological tools to efficiently provide for reconsent and related forms of specific consent.

### Conclusion

To be fair, legal controversies in research are surprisingly rare. However, experience tells us that consent can be a “life or death” issue for a biobank. If the proper protocols are not followed, the results of litigation can be devastating. For example, in 2010 the Texas Department of State Health Services was forced under settlement terms to destroy more than 5 million blood samples collected from newborn babies [77]. Five parents had sued over the failure to obtain consent [77]. Given the potential for this kind of well-publicized outcome, one would expect a more comprehensive response to the biobanking consent dilemma. However, to date, only a few jurisdictions have taken steps to explicitly resolve the legal challenges outlined above [78].

We believe the biobanking community needs to come to terms with both the reality that the types of consent used in biobanking often do not meet the requirements necessitated by relevant legal norms and, more importantly, that the there are numerous social forces and cultural trends that may be intensifying these unresolved consent issues. That said, we also understand the practical needs that drove the adoption of the modified consent strategies. Researchers, participants and institutions would all benefit from a defensible, sustainable and conceptually coherent consent policy. Given the rise in privacy concerns, the increased interest in rights of control, the rapid pace of technological development and the lack of consensus on preferred consent type, the time is now for policymakers and politicians to clear up the confusion.
